# Supplementary material for: Introducing an Innovative Pain Scale for Assessing Postpartum Pain in Mares: Preliminary Clinical Evaluation
Source: Animals (Basel). 2025 Nov 30;15(23):3454. doi: 10.3390/ani15233454 (PMC12691225; doi:10.3390/ani15233454)
Supplement: Supplementary file 1 [file animals-15-03454-s001.zip › Supplmentary Tables.pdf]

Table S1. The results of the online survey and Likert-type categorization of perceived ability.

Survey question	Yes (n)	No (n)	% Yes	Likert category*	Interpretation
Can you identify increased muscle tension in a mare by yourself?	244	171	58.8	2	Low–moderate perceived ability
Can you distinguish between a mare's natural whinnying and sounds of pain?	354	61	85.3	4	Very high perceived ability
Can you assess if a mare is sweating more than usual?	392	23	94.5	4	Very high perceived ability
Do you think that the position of the tail could indicate pain in the vaginal and abdominal region?	328	87	79.0	3	High perceived ability
Is observing facial expressions after parturition important, such as wrinkles indicating pain?	323	92	77.8	3	High perceived ability
Can you accurately count the number of breaths a mare takes in 60 seconds?	304	111	73.3	3	High perceived ability
Can you take the mare's rectal temperature?	288	127	69.4	2	Low–moderate perceived ability
Do you believe that using graphic visualization, such as illustrative pictures, significantly enhances the evaluation?	351	64	84.6	3	High perceived usefulness of visual aids
Do you believe you can independently assess the temperature of the hoof capsule (excessive heat)?	282	133	67.9	2	Low–moderate perceived ability
Can you determine a digital pulse without veterinary interference?	177	238	42.7	1	Very low perceived ability
Can you recognize when a mare's udder temperature is too high after parturition?	251	164	60.5	2	Low–moderate perceived ability

Can you accurately evaluate swelling in specific body parts, such as the udder and the abdomen?	385	30	92.8	4	Very high perceived ability
Can you assess whether the mare is defecating correctly?	355	60	85.5	4	Very high perceived ability

*1 = very low (<50% Yes); 2 = low–moderate (50–69.9% Yes); 3 = high (70–84.9% Yes); 4 = very high (≥85% Yes).

Table S2. Summary statistics and convergence diagnostics for the posterior distributions of parameters in the Bayesian linear mixed model relating pain score to salivary cortisol concentration.

number	parameter	median	pd	q2.5	q97.5	Rhat	ESS_{bulk}	ESS_{tail}
1	Intercept	344.75	1.000	222.21	450.19	<1.001	21,002	28,142
2	Pain_score	12.98	0.984	1.38	29.65	<1.001	13,933	17,047
3	b[(Intercept) mare_id:M_1]	0.07	0.507	-19.52	23.66	<1.001	28,317	26,570
4	b[Pain_score mare_id:M_1]	-1.80	0.711	-13.58	5.98	<1.001	14,231	20,114
5	b[(Intercept) mare_id:M_2]	-0.02	0.502	-22.11	22.00	<1.001	29,534	28,821
6	b[Pain_score mare_id:M_2]	-2.17	0.721	-16.26	5.93	<1.001	12,727	17,795
7	b[(Intercept) mare_id:M_3]	-0.26	0.528	-24.23	18.02	<1.001	30,890	28,837
8	b[Pain_score mare_id:M_3]	0.94	0.612	-8.30	13.34	<1.001	24,380	26,483
9	b[(Intercept) mare_id:M_4]	-0.22	0.524	-23.17	19.41	<1.001	34,583	29,931
10	b[Pain_score mare_id:M_4]	0.36	0.548	-10.09	12.28	<1.001	27,041	27,526
11	b[(Intercept) mare_id:M_5]	0.39	0.538	-21.63	25.12	<1.001	29,949	30,220
12	b[Pain_score mare_id:M_5]	3.20	0.762	-5.83	24.14	<1.001	19,407	28,804
13	sigma	73.11	1.000	48.81	121.48	<1.001	24,881	25,091
14	Sigma[mare_id:(Intercept),(Intercept)]	41.64	1.000	0.69	708.70	<1.001	17,997	23,061
15	Sigma[mare_id:Pain_score,(Intercept)]	-0.25	0.525	-140.64	103.17	<1.001	19,761	26,284
16	Sigma[mare_id:Pain_score,Pain_score]	31.36	1.000	0.65	366.43	<1.001	13,949	22,894

number—number of the model parameter (for reference to the rank plots, Sup. Fig. 3);

parameter—name of the parameter: Intercept, overall model intercept; Pain_score, coefficient (slope) for the increase in cortisol level for a one unit increase in pain score; b indicates a random intercept or slope for one of the mares (M_1 to M_5); sigma, residual standard deviation; Sigma[mare_id:(Intercept),(Intercept)], variance of random intercepts; Sigma[mare_id:Pain_score,(Intercept)], covariance between random slopes and random intercepts; Sigma[mare_id:Pain_score,Pain_score], variance of random slopes

median—median value of the draws from the posterior distribution (i.e. the best estimate of the value of the parameter)

pd—probability of direction (i.e. posterior probability that a positive parameter value is greater than zero or that a negative one is less than zero)

q2.5, q97.5—limits of the 95% equal-tailed credible interval for each parameter

Rhat—split potential scale reduction factor (values below 1.01 indicate convergence of the Markov chains used by the modeling algorithm) (Vehtari et al., 2021 [58])

ESS_{bulk}—estimate of effective number of samples from the center of the posterior distribution after accounting for correlation between samples (Vehtari et al., 2021 [58])

ESS_{tail}—estimate of effective number of samples from the tails of the posterior distribution after accounting for correlation between samples (Vehtari et al., 2021 [58])
