# Supplementary figures and images for: Introducing an Innovative Pain Scale for Assessing Postpartum Pain in Mares: Preliminary Clinical Evaluation
Source: Animals (Basel). 2025 Nov 30;15(23):3454. doi: 10.3390/ani15233454 (PMC12691225; doi:10.3390/ani15233454)

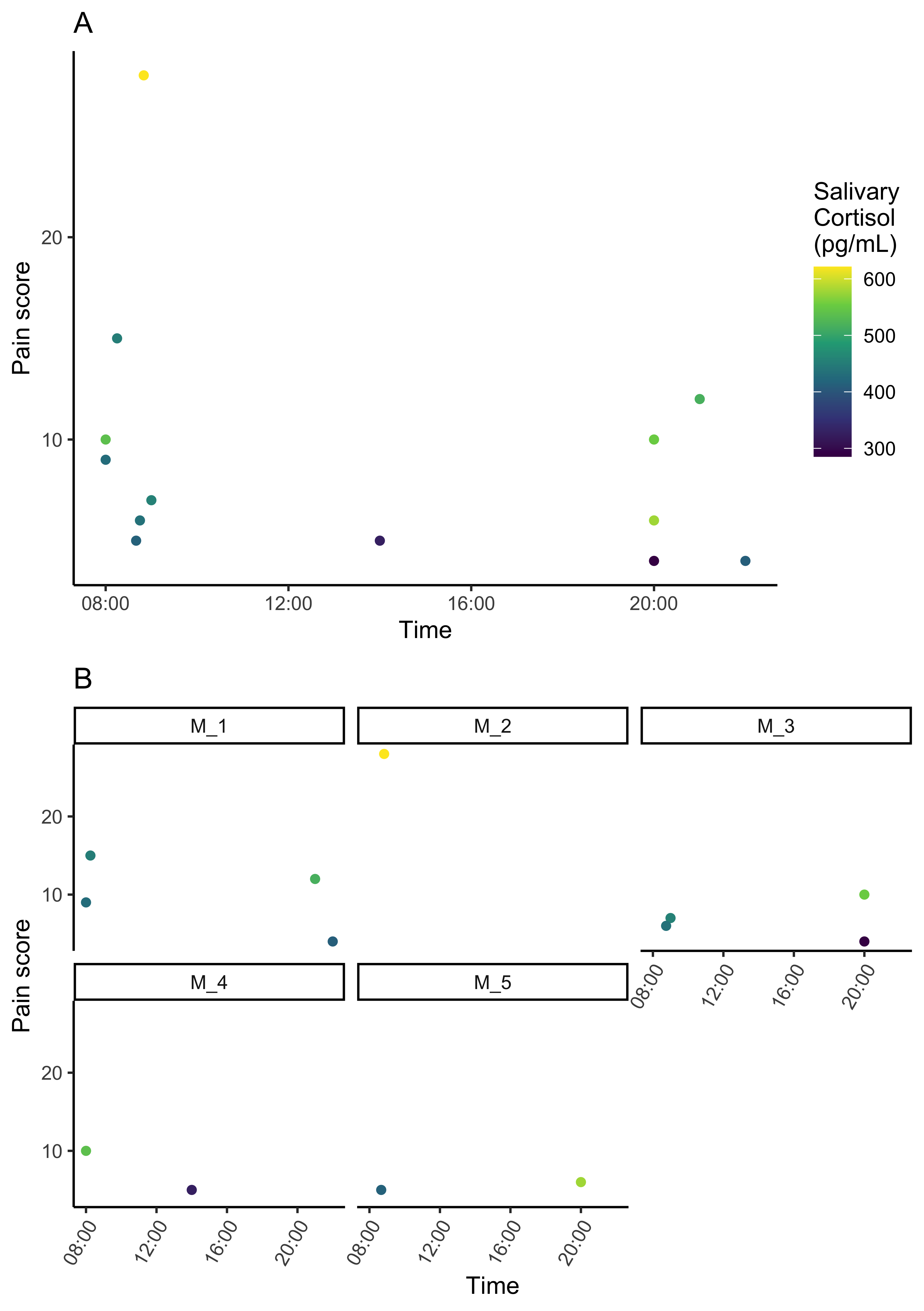

Supplement: Supplementary file 1 [file animals-15-03454-s001.zip › Supplmentary Figure S1.tiff]

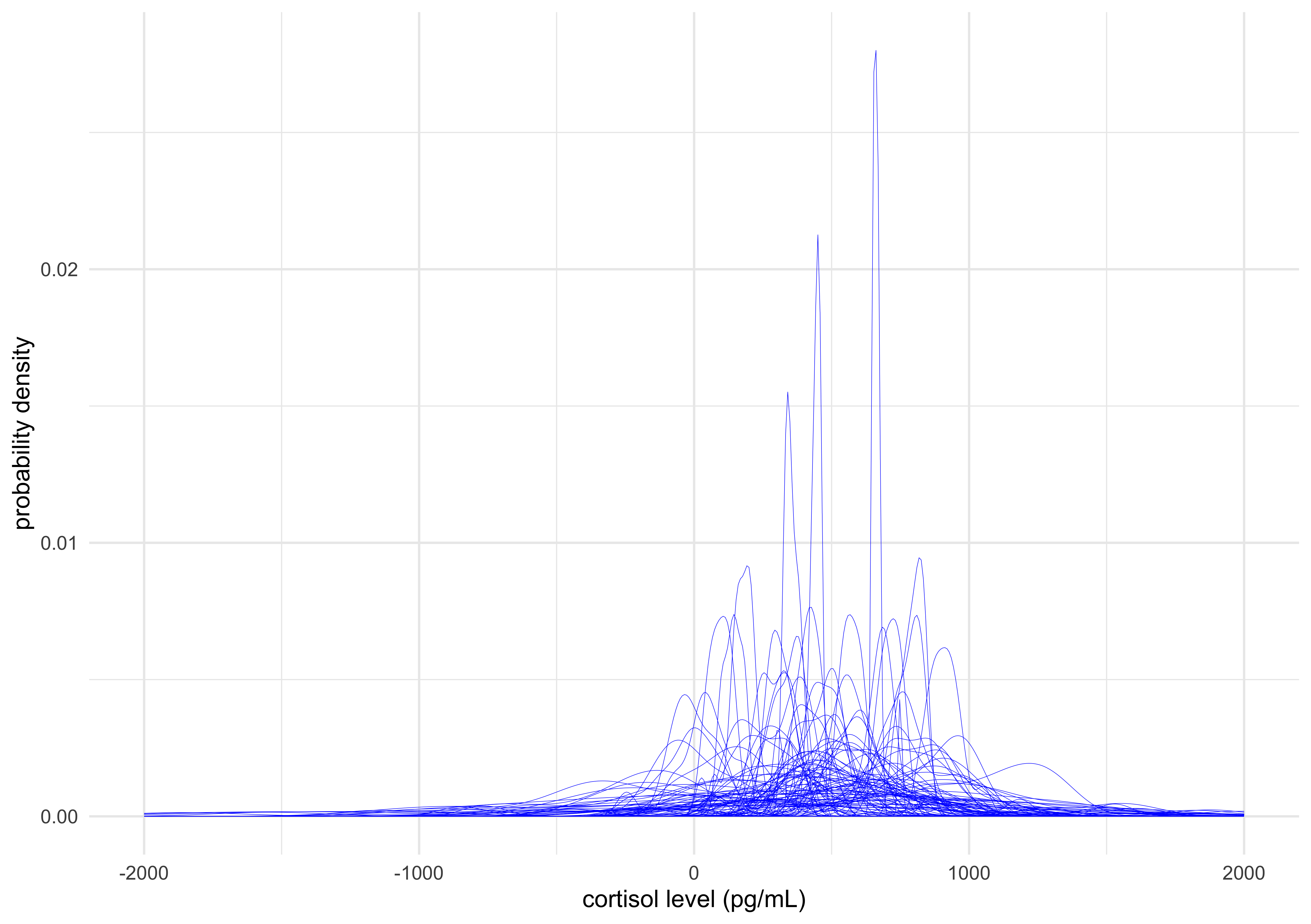

Supplement: Supplementary file 1 [file animals-15-03454-s001.zip › Supplmentary Figure S2.tiff]

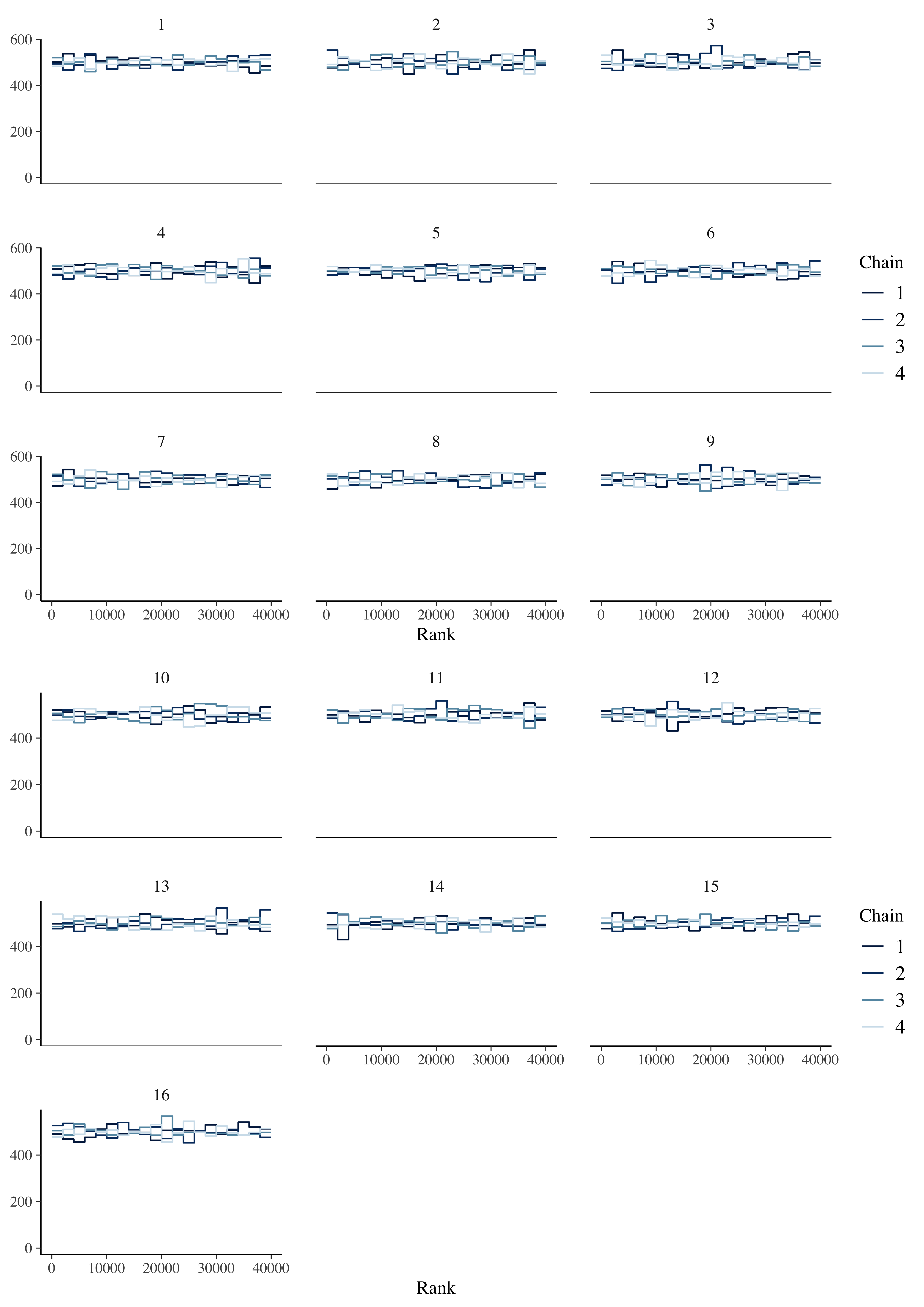

Supplement: Supplementary file 1 [file animals-15-03454-s001.zip › Supplmentary Figure S3.tiff]

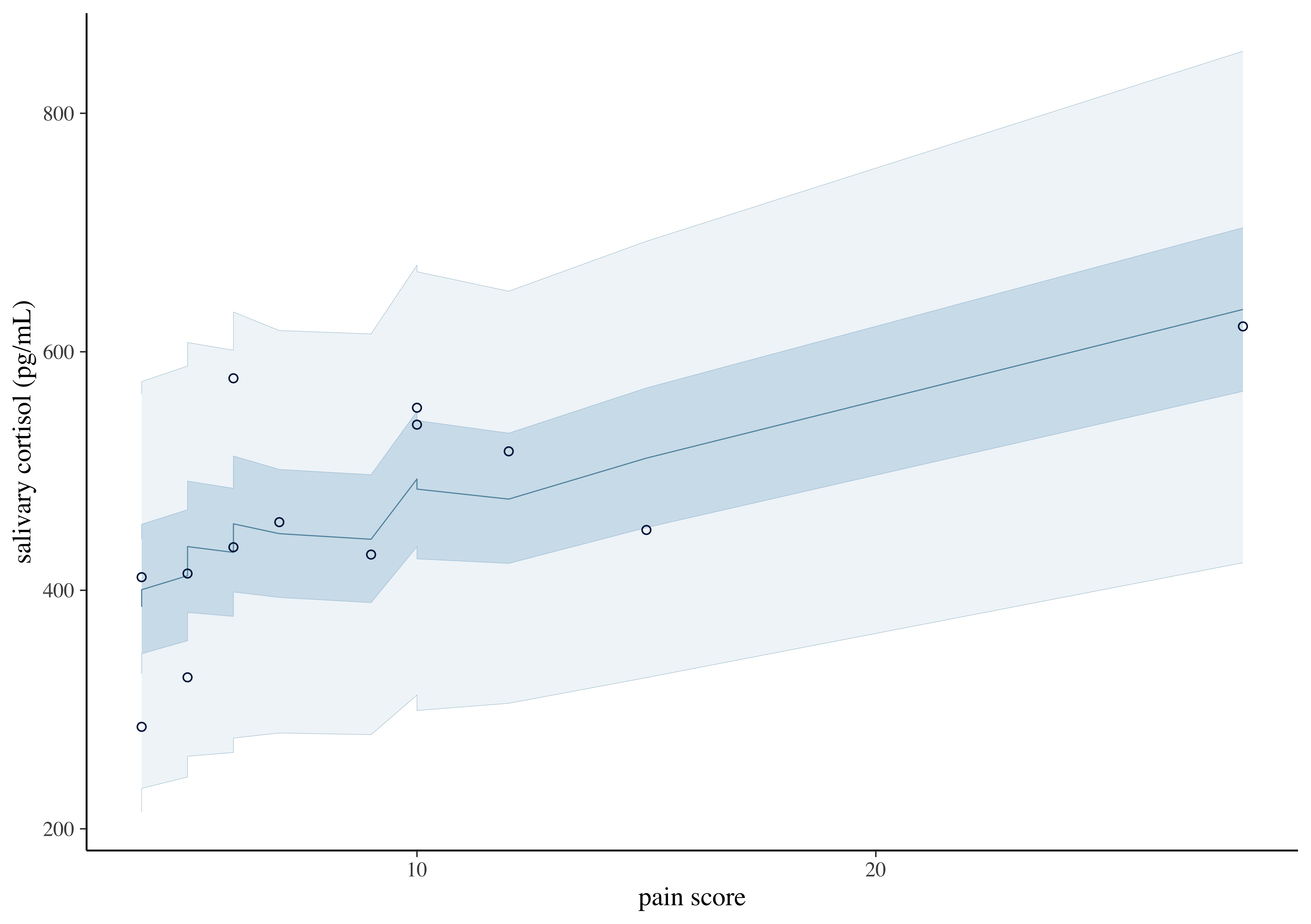

Supplement: Supplementary file 1 [file animals-15-03454-s001.zip › Supplmentary Figure S4.tiff]
